# 3′-Deoxy-3′-[^18^F]-Fluorothymidine PET Imaging Reflects PI3K-mTOR-Mediated Pro-Survival Response to Targeted Therapy in Colorectal Cancer

**DOI:** 10.1371/journal.pone.0108193

**Published:** 2014-09-23

**Authors:** Eliot T. McKinley, Ping Zhao, Robert J. Coffey, M. Kay Washington, H. Charles Manning

**Affiliations:** 1 The Vanderbilt University Institute of Imaging Science (VUIIS), Vanderbilt University Medical School, Nashville, TN, United States of America; 2 Department of Biomedical Engineering, Vanderbilt University Medical School, Nashville, TN, United States of America; 3 Department of Medicine, Vanderbilt University Medical School, Nashville, TN, United States of America; 4 Department of Vanderbilt Ingram Cancer Center, Vanderbilt University Medical School, Nashville, TN, United States of America; 5 Department of Pathology, Vanderbilt University Medical School, Nashville, TN, United States of America; 6 Department of Radiology and Radiological Sciences, Vanderbilt University Medical School, Nashville, TN, United States of America; 7 Department of Neurosurgery, Vanderbilt University Medical School, Nashville, TN, United States of America; 8 Department of Chemical and Physical Biology, Vanderbilt University Medical School, Nashville, TN, United States of America; University of Kentucky, United States of America

## Abstract

Biomarkers that predict response to targeted therapy in oncology are an essential component of personalized medicine. In preclinical treatment response studies that featured models of wild-type *KRAS* or mutant *BRAF* colorectal cancer treated with either cetuximab or vemurafenib, respectively, we illustrate that [^18^F]-FLT PET, a non-invasive molecular imaging readout of thymidine salvage, closely reflects pro-survival responses to targeted therapy that are mediated by PI3K-mTOR activity. Activation of pro-survival mechanisms forms the basis of numerous modes of resistance. Therefore, we conclude that [^18^F]-FLT PET may serve a novel and potentially critical role to predict tumors that exhibit molecular features that tend to reflect recalcitrance to MAPK-targeted therapy. Though these studies focused on colorectal cancer, we envision that the results may be applicable to other solid tumors as well.

## Introduction

With increased ability to rapidly and inexpensively characterize the genetic basis of an individual patient's tumor, personalized therapies are rapidly becoming widespread in oncology. Landmark examples of the success of personalized medicine in oncology include the use of vemurafenib to treat *^V600E^BRAF* melanoma [1] and trastuzumab to treat *HER2* overexpressing breast cancers [2]. With an increasing reliance on molecularly targeted therapies, there remains an equally critical challenge to develop and validate specific biomarkers that reflect target inhibition, pathway inactivation, and predict overall clinical response. Most biomarkers utilized in oncology studies require tissue sampling which is highly susceptible to sampling error and bias due to heterogeneity. Serum-based biomarkers lack the ability to directly visualize the tumor and demonstrate that the measured effect is directly the result of tumor response. Non-invasive imaging circumvents these limitations and offers major advantages over traditional biomarkers. Of the imaging modalities available clinically, the sensitivity and the ability to readily produce biologically active molecules bearing positron-emitting isotopes makes positron emission tomography (PET) one of the most attractive modalities for detecting tumors and profiling biological responses to therapy.

Our laboratory has studied the biological basis of 3′-deoxy-3′[18F]-fluorothymidine ([^18^F]-FLT) accumulation in tumors [3]–[6] and other diseased tissue [7]. A thymidine analog, [^18^F]-FLT was originally developed to serve as a non-invasive measure of cellular proliferation, with obvious utility in oncology [8], [9] by reporting on the thymidine salvage pathway that provides DNA precursors to dividing cells. Upon cellular internalization, [^18^F]-FLT is phosphorylated in a reaction catalyzed by the cytosolic enzyme thymidine kinase 1 (TK1) and trapped in the cell. TK1 activity is closely correlated with DNA synthesis and tends to be diminished in quiescent cells. [^18^F]-FLT has been widely studied as a marker of treatment response in a spectrum of tumor types and treatments both in the pre-clinical and clinical settings [10]. However, it is important to note that unlike more generalizable proliferation markers, such as Ki67, [^18^F]-FLT PET reflects proliferative indices to variable and potentially unreliable extents [6], [11]. [^18^F]-FLT-PET cannot discriminate moderately proliferative, thymidine salvage-driven tumors from those of highly proliferative tumors that rely primarily upon *de novo* thymidine synthesis. Despite a lack of correlation with proliferation in some circumstances, we envisioned that TK1 levels, and thus [^18^F]-FLT PET, could reflect other potentially important molecular events associated with response to therapy.

Using preclinical models of colorectal cancer we demonstrate two circumstances where [^18^F]-FLT PET does not correlate with proliferation, but rather reflects PI3K-mTor mediated pro-survival responses to targeted therapy. In these settings, [^18^F]-FLT PET was discordant 2-deoxy-2-[^18^F]fluoro-D-glucose ([^18^F]-FDG) PET, the most widely utilized tracer in clinical oncology, which was not sensitive to mTOR- or PI3K-pathway activity. Cetuximab mediated inhibition of MAPK activity in a wild-type *KRAS* cell line model and vemurafenib-mediated inhibition of BRAF in a *^V600E^BRAF* mutant cell line model had no effect on [^18^F]-FLT PET unless PI3K-mTOR was subsequently attenuated pharmacologically or *via* genetic silencing. Overall, these studies demonstrate a novel role for [^18^F]-FLT PET as a means to predict tumors that resist MAPK inhibition through PI3K-mTOR activation in colorectal cancer and potentially other solid tumors.

## Materials and Methods

### Cell lines and mouse models

All studies were approved by the Vanderbilt University Institutional Animal Care and Use Committee and all efforts were made to minimize animal suffering. DiFi human cells were a gift from Dr. Bruce Boman [12] and COLO 205 cells were obtained from ATCC (CCL-222). DiFi human colorectal cancer cells were grown in Dulbecco's modified Eagle's medium (DMEM) and COLO 205 cells were grown in RPMI (Cellgro) with 10% fetal bovine serum, (Atlanta biologicals), 1% penicillin and streptomycin (GIBCO) at 37°C and 5% CO_2_. C225 was obtained from the Vanderbilt Pharmacy, PLX4032 and PLX4720 were synthesized as described [13], PP242 was obtained from Sigma Aldrich, and BEZ235 from Selleck Chem. Stock solutions of each drug were prepared and aliquoted to achieve final drug concentrations for *in vitro* studies.

For *in vivo* studies, cell line xenografts were generated in athymic nude mice (Harlan) as described [3] and treatment began when volume reached approximately 150 mm^3^. For treatment of DiFi xenografts, saline vehicle, 20 mg/kg, or 40 mg/kg cetuximab were administered i.p. every third day. PET imaging of DiFi xenograft bearing mice was conducted 7 days after the initiation of treatment, 24 hours following the third treatment. For COLO 205 xenografts, mice were treated with DMSO vehicle, 60 mg/kg PLX4720, or 40 mg/kg BEZ235 daily by oral gavage (100 µL total volume). PET imaging of COLO 205 xenograft bearing mice was conducted 4 days after the initiation of treatment, approximately 24 hours following the third treatment. Mice were sacrificed immediately following completion of PET imaging.

### siRNA methods

Raptor (L-004107-00) and random sequence (D-001810-01) siRNA reagents were obtained from Thermo Scientific. siRNA was transfected into DiFi cells using the DharmaFect transfection kit (Thermo Scientific) as per manufacturer's instructions. In short, 500,000 DiFi cells were plated into each well of a 6-well plate. After 24 hours, siRNA was added to the appropriate wells. After 48 hours, saline vehicle, 0.5 µg/mL or 5.0 µg/mL C225 were added to the appropriate wells. After a further 24 hours, cells were harvested for Western blotting and qRT-PCR as described below.

### Radiopharmaceutical synthesis

[^18^F]-FLT was prepared in a two-step, one-pot reaction as described [4], [14]. [^18^F]-FLT was obtained with average radiochemical purity of 98.3% and specific activity ≥345.5 TBq/mmol. [^18^F]-FDG was synthesized in the Vanderbilt University Medical Center Radiopharmacy and distributed by PETNET. The average radiochemical purity of the product was 98.5% and specific activity was more than 37 TBq/mmol.

### Small-animal imaging

Small-animal PET imaging was performed using a dedicated microPET scanner (Concorde Microsystems Focus 220). Mice were maintained under 2% isofluorane anesthesia in oxygen at 2 L/min and kept warm via a circulating water heating pad for the duration of the PET scan. For [^18^F]-FLT PET imaging animals were administered 7.4–9.3 MBq (200–250 µCi) intravenously. Animals were allowed free access to food and water during a 40 minute uptake period, followed by anesthetization and a 20 minute image acquisition. For [^18^F]-FDG PET imaging, mice were fasted for approximately 6 h prior to imaging and warmed in a heated (31°C) chamber for 1 h prior to [^18^F]-FDG injection and during the uptake period to minimize brown fat uptake of [^18^F]-FDG. Mice were administered 7.4–9.3 MBq of [^18^F]-FDG intravenously and allowed free access to water during a 50 minute uptake period followed by a 10 min PET acquisition.

PET data were reconstructed using OSEM3D/MAP. The resulting three-dimensional reconstructions had an x-y voxel size of 0.474 mm and inter-slice distance of 0.796 mm. ASIPro software (Siemens) was used to manually draw three-dimensional regions of interest in the tumor volume. Tumor samples were immediately collected following [^18^F]-FLT-PET and flash frozen in liquid nitrogen. [^18^F]-FLT uptake was quantified as the percentage of the injected dose per gram of tissue (%ID/g) by dividing the ROI activity by the injected dose and multiplying by 100.

### Immunoblotting

For *in vitro* studies, cells were lysed with CelLytic M (Sigma Aldrich), centrifuged at 16,000 rpm for 15 minutes at 4°C, and the supernatant removed prior to measurement of protein concentration by bicinchoninic acid (BCA) assay (Thermo Scientific). For *in vivo* studies, fresh frozen tissue was homogenized in CelLytic MT (Sigma Aldrich) lysis buffer, centrifuged at 16,000 rpm for 15 minutes at 4°C, and the supernatant removed prior to measurement of protein concentration by BCA assay. Prior to resolution by electrophoresis, 20–40 µg of protein from each sample was loaded into 7.5–12% SDS PAGE gels and transferred to PVDF membranes (PerkinElmer). Membranes were blocked overnight at 4°C in tris-buffered saline 0.1% Tween-20 (TBST) containing 5% w/v nonfat dry milk powder. Subsequently, membranes were interrogated with antibodies obtained from Cell Signaling Technology unless noted to p-ERK 1/2 Thr202/Tyr204 (#4370), ERK 1/2 (#4372), TK1 (Abcam, #57757), p27 (#3686), p-AKT Ser473 (#4060), p-AKT Thr308 (#4056), AKT (#4685), p-rpS6 Ser236/236 (#4858), rpS6 (#2217), p-4EBP1 Thr37/46 (#2855), p-MEK Ser217/221 (#9154), MEK (#9126), DUSP6 (#3058), β-actin (#4970), β-tubulin (Novus Biologicals, #NB600-936). Membranes were probed for 1 hour at room temperature in TBST with 3% bovine serum albumin (BSA). Membranes were subsequently incubated for 1 hour at room temperature with horseradish peroxidase-conjugated secondary antibody (Jackson ImmunoResearch) diluted 1∶5000 in TBST containing 3% BSA. Western Lightning Plus-ECL (PerkinElmer) was used for chemiluminescent detection on a Xenogen IVIS 200.

### Immunohistochemistry (IHC)

Animals were sacrificed and tumor samples were collected immediately following PET imaging, then subsequently fixed in 10% formalin for 24 hours. Tissues were then transferred to 70% ethanol prior to paraffin embedding. Tissues were sectioned (5 µm thickness) and stained for p-rpS6 (Cell Signaling, #4858, 1∶100 primary dilution), p-AKT Ser473 (Cell Signaling, #4060, 1∶100 primary dilution), Ki67 (Dako, #M7240, 1∶100 primary dilution), p27 (Dako, #M7203, 1∶100 primary dilution). Briefly, the tissue samples were de-paraffinized, rehydrated, and antigen retrieval was performed using citrate buffer (ph 6.0) solution for 15 minutes at 105°C followed by a 10 minute bench cool down. The samples were then treated with 3% hydrogen peroxide to eliminate endogenous peroxidase activity. The sections were subsequently blocked with a serum-free protein blocking reagent for 20 minutes. Primary antibody detection was accomplished using the following system: The tissue sections were incubated at room temperature for 60 minutes at the noted dilutions followed by a 30 minute incubation utilizing the Envision + System-HRP Labeled Polymer detection method (Dako, Carpinteria, CA). Staining was completed after incubation with a 3,3′-Diaminobenzidine substrate-chromogen solution. Tissue slides were imaged at 40x magnification and manually scored to determine the percentage of positive cells per high power field.

### qRT-PCR

RNA was collected by using RNeasy as suggested by supplier (QIAGEN). Both cDNA and realtime PCR experiments were carried out by using iScript cDNA synthesis kit and iQ SYBR Green Supermix (BIO-RAD) by supplier instruction. Amplifications were performed in a BIO-RAD CFX96 Real-Time System for 40 cycles. Data was acquired by Bio-Rad CFX Manager software and fold changes were analyzed as described by Schefe et al [15]. Human TK1 (5′-AATCAGCTGCATTAACCTGCCCAC-3′ forward; 5′-ATCACCAGGCACTTGTACTGAGCA-3′ reverse), human P27 (5′-AGCAATGCGCAGGAATAAGGAAGC-3′ forward; 5′-TACGTTTGACGTCTTCTGAGGCCA-3′ reverse) were obtained from Integrated DNA Technologies.

### Statistical Analysis

Statistical significance of data was evaluated using the non-parametric Wilcoxon Rank Sum (Mann-Whitney U) tests using the GraphPad Prism 4 software package. Differences were considered statistically significant if p<0.05.

## Results

### Regulation of TK1 following EGFR blockade in wild-type *KRAS* colorectal cancer cells

**Figure 1:**
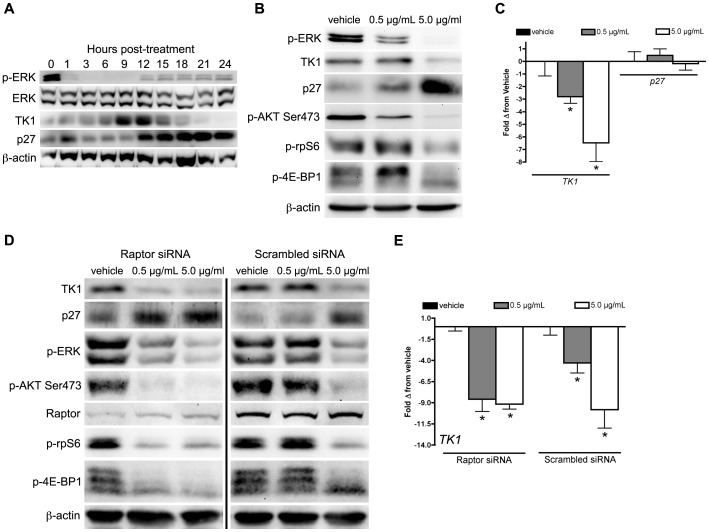
Diminished TK1 protein levels correlate with attenuation of mTOR-PI3K pathway activity and upregulation of p27. (**A**) Western blot of DiFi cell lysates following cetuximab exposure (5 µg/mL) resulted in rapid attenuation of downstream MAPK targets including p-ERK, which remained well-below baseline levels to 24 hours. Paradoxically, TK1 protein levels increased from 1–12 hours until p27 protein levels rose, at which time TK1 fell bellow baseline. (**B**) DiFi cells treated with either 0.5 µg/mL or 5.0 µg/mL cetuximab resulted in a 50% reduction and full attenuation of p-ERK protein levels, respectively at 24 hours. At 0.5 µg/mL TK1 levels were unaffected despite a modest rise in p27 protein levels. However, 5.0 µg/mL cetuximab resulted in greatly decreased TK1 with a large increase in p27 protein levels. PI3K-mTOR activity, as measured by p-AKT Ser473, p-rpS6, and p-4E-BP1, was either maintained or elevated at 0.5 µg/mL cetuximab but was attenuated at 5.0 µg/mL cetuximab. (**C**) qRT-PCR analysis showed *TK1* mRNA was significantly reduced at 0.5 µg/mL (p = 0.0279) and 5.0 µg/mL (p = 0.0186), while no change in *p27* mRNA levels was observed at either dose. (**D**) Silencing mTORC1 facilitated upregulation of p27 and attenuated TK1 protein levels at 0.5 µg/mL cetuximab without affecting the anti-MAPK activity effects of cetuximab. Levels of p-AKT Ser473, p-rpS6, and p-4E-BP1 were reduced at 0.5 µg/mL cetuximab exposure with Raptor knockdown but not in scrambled siRNA control. (**E**) As evidence of the functionality of p27, *TK1* mRNA levels were similarly reduced at 0.5 µg/mL and 5.0 µg/mL with Raptor knockdown.

Previously, we showed that imaging readouts of EGFR occupancy and apoptosis, but not [^18^F]-FLT PET, predicted response to cetuximab in DiFi cell line xenografts that express wild-type KRAS and exhibit EGFR amplification [3], [12]. Therefore, in this study, we first sought to elucidate the relationships between EGFR blockade with cetuximab and TK1 regulation. Initially, we used cultured DiFi cells to evaluate the temporal relationship between cetuximab exposure and p-ERK, TK1, and p27 protein levels over 24 hours (**Fig. 1A**). As expected, p-ERK levels were almost immediately attenuated, showing a dramatic reduction within the first hour of cetuximab exposure (5.0 µg/mL), and remaining well-below baseline levels for up to 24 hours. Paradoxically, TK1 levels increased following cetuximab exposure, which peaked at 12 hours, and then rapidly declined to below baseline levels. Protein levels of the cell cycle inhibitor p27 rose dramatically and plateaued by 12 hours. The inverse relationship between TK1 and p27 protein levels implicated p27 as a key determinant of TK1 regulation in DiFi cells.

To further evaluate the relationships between MAPK pathway inhibition, TK1 regulation, and p27, DiFi cells were exposed to two concentrations (0.5 µg/mL and 5.0 µg/mL) of cetuximab for 24 hours (**Fig. 1B**). At 0.5 µg/mL, TK1 protein levels were unaffected despite modestly elevated p27 and an approximately 50% reduction of p-ERK and p-AKT Ser473. Conversely, the 5.0 µg/mL level of exposure resulted in dramatically attenuated TK1 levels. Similar to the time course study, reduced TK1 correlated with a robust increase in p27. Additionally, complete attenuation of p-ERK and p-ATK Ser473 was observed at 5.0 µg/mL. In contrast to protein, *TK1 mRNA* tended to follow p-ERK levels more directly (**Fig. 1C**), which is not surprising given *TK1*'s dependence on E2F transcription [16], a downstream product of MAPK activity. Interestingly, *p27* mRNA was unaffected by cetuximab exposure, suggesting that elevated p27 protein levels stemmed from inhibition of downstream targets that affect post-transcriptional modification of p27, with AKT typically being one of these [17]. To determine if TK1 protein levels observed at 0.5 µg/mL levels of exposure were maintained through increased translational efficiency, we measured p-rpS6 and p-4E-BP1 levels, both products of pro-translational PI3K-mTOR activity (**Fig. 1B**). We found p-rpS6 levels to be attenuated only at the highest concentration of cetuximab. Furthermore, p-4E-BP1 levels were elevated at the lower concentration of cetuximab, suggesting increased potential for translation at the ribosomal cap [18]. Taken together with the *TK1* mRNA levels, these results may suggest that translational efficiency of TK1 at the lower level of cetuximab exposure that likely proceed through activation of mTOR.

### Silencing mTOR-PI3K activity facilitates cetuximab-mediated attenuation of TK1 levels in DiFi cells

To explore the role of mTOR on TK1 regulation in this context, we silenced mTOR using siRNA to Raptor, an essential member of the functional mTOR Complex 1 (mTORC1) [19]. In the absence of mTORC1 activity, TK1 protein levels were attenuated at both 0.5 µg/mL and 5.0 µg/mL concentrations of cetuximab, without an obvious effect on MAPK pathway activity (**Fig. 1D**). As expected, Raptor silencing resulted in greater cetuximab-mediated attenuation of p-AKT Ser473 and p-rpS6 as well as blocking phosphorylation of 4E-BP1. In addition to attenuated TK1 protein levels, mTORC1 inactivation resulted in increased p27 protein levels that appeared to be at least partially responsible for *TK1* transcriptional regulation at the lowest level of cetuximab exposure (**Fig. 1E**). Interestingly, as observed previously, the rise in p27 protein levels was itself not a product of increased *p27* transcription (**Fig. S1**). These results suggest that mTOR activation imparts its effect on TK1 through downstream effectors such as AKT and ERK through post-translational modification and degradation of cell cycle inhibitors, including p27 [17]. As evidence of this, mTORC1 inhibition in conjunction with EGFR blockade led to a profound reduction of TK1 protein levels not observable with cetuximab exposure alone at the lower concentration.

### [^18^F]-FLT PET reflects inhibition of PI3K-mTOR activity in cetuximab treated DiFi cell line xenografts *in vivo*

**Figure 2:**
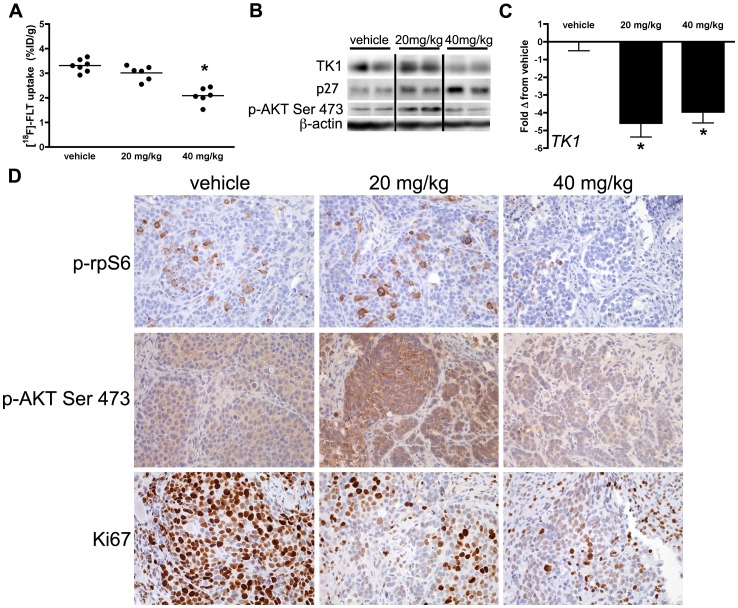
[^18^F]-FLT PET reflects inhibition of PI3K-mTOR activity in cetuximab-treated DiFi xenografts. DiFi tumor xenografts were imaged on day 7 of 20 mg/kg or 40 mg/kg cetuximab treatment regimens. (**A**) [^18^F]-FLT PET was only reduced in DiFi xenografts treated at the 40 mg/kg level (p = 0.0012). (**B**) Western blot analysis of tissues harvested immediately after imaging confirmed that TK1 protein levels were only decreased at the 40 mg/kg dose level. Increased p27 protein levels were observed at the 40 mg/kg dose levels. Increased p-AKT Ser473 protein levels were observed at the 20 mg/kg dose level. (**C**) Similar to *in vitro* observations, *TK1* mRNA was significantly reduced at both 20 mg/kg (p = 0.0009) and 40 mg/kg (p = 0.0001) dose levels. (**D**) IHC analysis of p-rpS6 and p-AKT Ser473 showed slightly elevated staining levels at 20 mg/kg. However, both markers were greatly reduced at 40 mg/kg. Ki67 immunoreactivity was reduced at both levels of cetuximab exposure.

We subsequently imaged DiFi xenograft-bearing mice with [^18^F]-FLT PET following cetuximab treatment (20 mg/kg or 40 mg/kg) or saline vehicle. In agreement with our previous studies [3], similar [^18^F]-FLT accumulation was observed in xenografts treated with vehicle or 20 mg/kg cetuximab. Increasing the dosage of cetuximab to 40 mg/kg resulted in diminished [^18^F]-FLT accumulation (**Fig. 2A**). Diminished TK1 protein levels were observed at the 40 mg/kg dosage relative to vehicle- and 20 mg/kg cetuximab-treated tumors (**Fig. 2B**). Similar to *in vitro* studies, diminished TK1 protein levels correlated with elevated p27 protein, but not *p27* mRNA levels (**Fig. S2**). Additionally, p-AKT Ser473 tumor protein levels were increased at the 20 mg/kg dose compared to both vehicle and 40 mg/kg cetuximab. TK1 protein levels were unaffected by the lower dosage of cetuximab, despite significantly reduced *TK1* mRNA (**Fig. 2C**), which was reduced relative to vehicle-treated controls at both levels of cetuximab exposure. IHC of imaging-matched xenograft tissues illustrated that PI3K-mTOR activity, as measured by p-rpS6 and p-AKT Ser473 levels, was inhibited at the highest cetuximab exposure (**Fig. 2D** **, Fig. S3**). Interestingly, Ki67 was reduced at both levels of cetuximab exposure (**Fig 2D** **, Fig. S4**), suggesting that [^18^F]-FLT PET was decoupled from standard measures of proliferation at the lower dosage. While [^18^F]-FLT PET appeared to be sensitive to PI3K-mTOR activity, [^18^F]-FDG PET imaging was not sensitive to this phenomenon. We observed reduced [^18^F]-FDG uptake relative to vehicle controls at both levels of cetuximab exposure (**Fig. S5**). Overall, these results suggest that [^18^F]-FLT PET provides unique information regarding mTOR signaling that is not captured by [^18^F]-FDG PET or standard measures of proliferation, such as Ki67 IHC.

### TK1 protein levels do not reflect ^V600E^BRAF inhibition in COLO 205 cells

**Figure 3:**
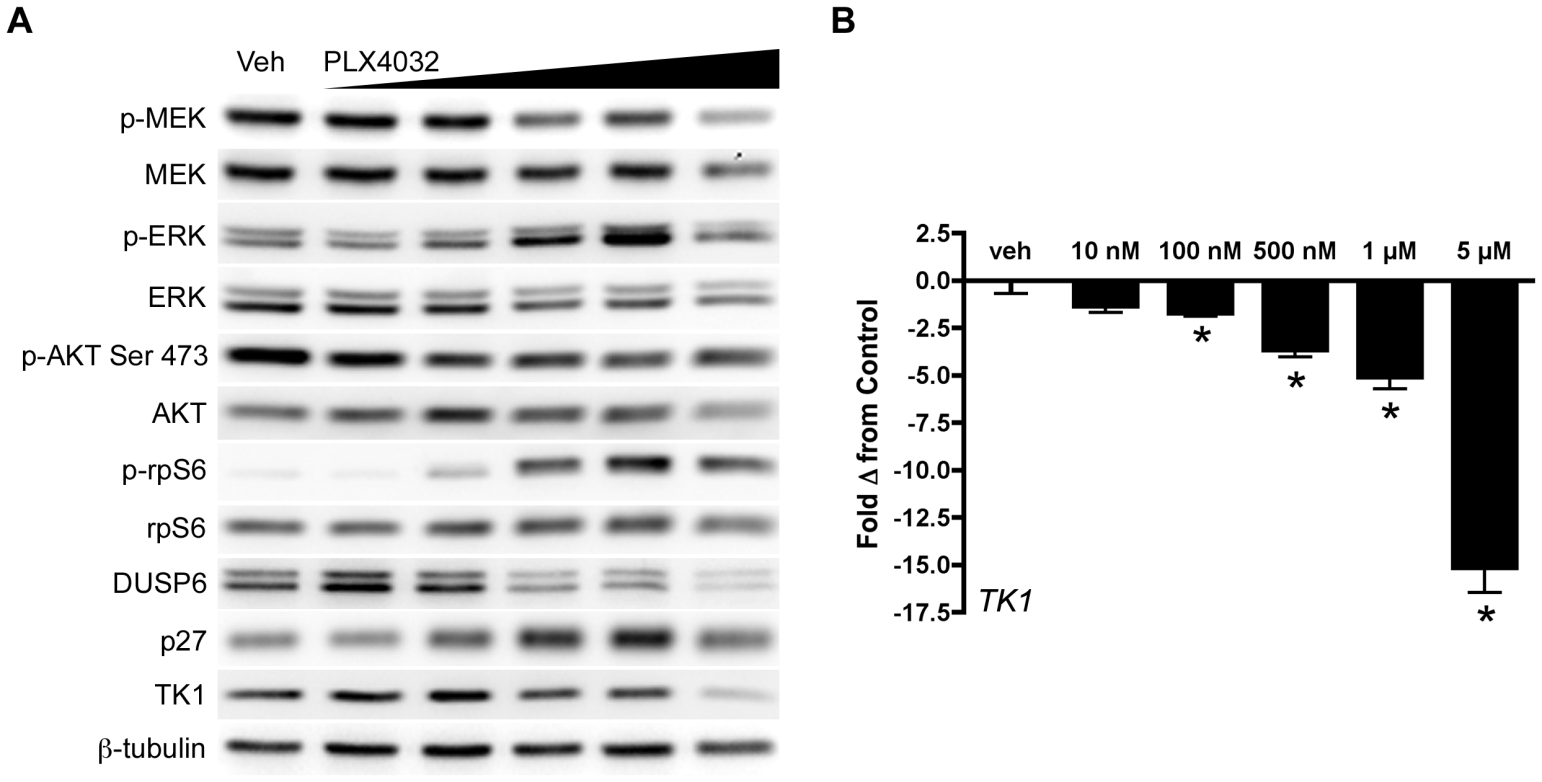
TK1 protein levels do not reflect p-ERK attenuation following inhibition of ^V600E^BRAF inhibition in COLO 205 cells. COLO 205 cells were collected 48 hours of PLX 4032 exposure at 10 nM, 100 nM, 500 nM, 1 µM, or 5 µM. (**A**) Western blot analysis demonstrated target inhibition of p-MEK despite increased p-ERK levels. PI3K-mTOR signaling was elevated in a PLX 4032-dependent manner as exhibited by a steady rise in p-rpS6 levels. The ERK-phosphatase DUSP6 decreased in conjunction with mTOR signaling and was inversely proportional to p-ERK levels. A slight increase in p27 levels were observed concomitantly with only modest changes in TK1 levels, except at the highest dose of PLX4032. (**B**) Decreased *TK1* mRNA levels were observed at all drug concentrations above 10 nM (p<0.05).

Analogous to the DiFi model treated with cetuximab, we evaluated relationships between target inhibition, downstream effectors, and TK1 levels in a ^V600E^BRAF expressing cell line, COLO 205, treated with a selective ^V600E^BRAF inhibitor (**Fig. 3**). PLX4032 exposure for 48 hours led to concentration-dependent inhibition of p-MEK levels. Paradoxically, p-ERK levels were increased in a concentration-dependent manner, except at the highest dose (5 µM) (**Fig. 3A**). The mismatch between p-MEK and p-ERK was not observed at exposure durations of 2 hours (**Fig. S6**) or 24 hours (**Fig. S7**). While p-AKT Ser473 levels were only moderately reduced with PLX4032 exposure at 48 hours, we observed a concentration-dependent increase in p-rpS6 that correlated with the increase in p-ERK. Concomitantly increased p-rpS6 and diminished DUSP6 levels suggested that the increase in p-ERK was at least partially resulted from diminished phosphatase activity that was likely related to mTOR activation [20]. PLX4032 exposure led to a modest increase in p27 protein levels. TK1 levels were increased slightly at lower PLX4032 concentrations and unchanged from vehicle at higher levels of exposure, except for the highest concentration (5 µM). Surprisingly, *TK1* mRNA levels appeared to be more closely associated with inhibition of p-MEK and, unlike TK1 protein levels, were reduced in an essentially concentration-dependent manner (**Fig. 3B**).

### [^18^F]-FLT PET, but not [^18^F]-FDG PET, reflects elevated mTOR activity and correlates with a lack of p-ERK inhibition in PLX4720-treated COLO 205 xenografts

**Figure 4:**
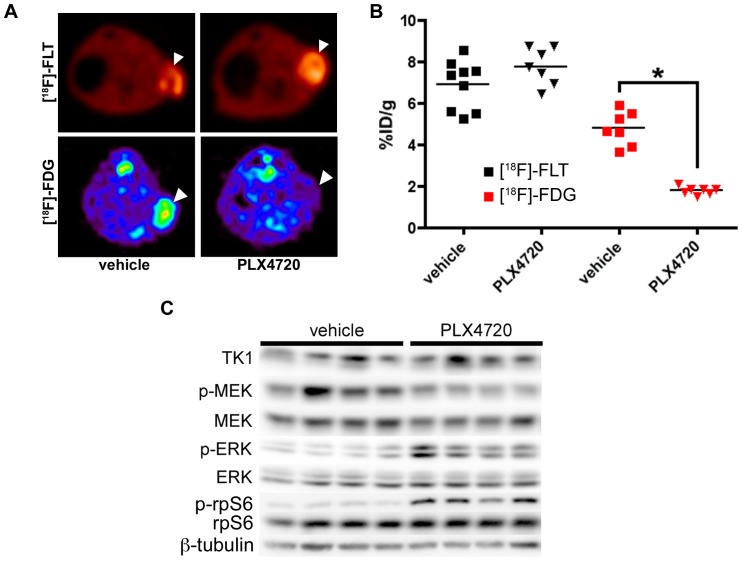
PLX4720 exposure does not affect [^18^F]-FLT PET in COLO 205 xenografts, despite evidence of target inhibition and diminished [^18^F]-FDG uptake. (**A**) Representative transverse [^18^F]-FLT and [^18^F]-FDG PET images acquired after three daily treatments with vehicle or 60 mg/kg PLX4720 (tumor indicated by arrowhead). (**B**) Quantification of PET data illustrated similar [^18^F]-FLT uptake in vehicle-treated and PLX4720-treated tumors. Unlike [^18^F]-FLT PET, PLX4720 exposure elicited a significant reduction in [^18^F]-FDG uptake (p = 0.0006). (**C**) Western blot analysis of vehicle- and PLX4720-treated tumor tissue confirmed that PLX4720 had no effect on TK1 protein levels in agreement with [^18^F]-FLT PET. Target inhibition as measured by p-MEK levels was observed. However, similar to *in vitro* studies, PLX4720-treated COLO 205 xenografts exhibited elevated p-ERK and p-rpS6 protein levels relative to vehicle controls.

Mice bearing COLO 205 xenografts were treated daily with 60 mg/kg PLX4720 for 4 days and imaged with [^18^F]-FLT PET or [^18^F]-FDG PET (**Fig. 4**). In agreement with *in vitro* studies showing that BRAF inhibition had little effect on TK1 levels in COLO 205 cells, treatment with PLX4720 had little effect on [^18^F]-FLT PET imaging. Conversely, [^18^F]-FDG PET was significantly reduced in similarly treated cohorts (**Fig. 4B** **)**. In agreement with imaging, TK1 levels of PLX4720-treated xenografts were similar to vehicle-treated controls. Also similar to *in vitro* studies, we found elevated p-ERK levels and p-rpS6 levels in PLX4720 treated xenografts relative to vehicle-treated controls, despite inhibition of the BRAF effector, p-MEK.

### Regulation of TK1 following mTOR or dual PI3K-mTOR inhibition and ^V600E^BRAF inhibition in COLO 205 cells

**Figure 5:**
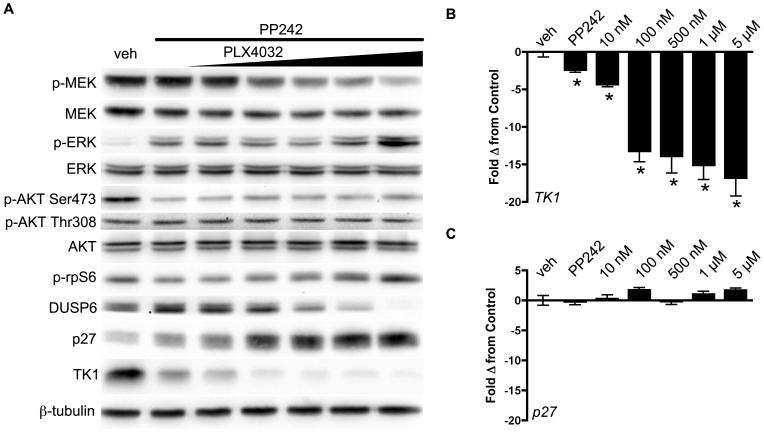
Combined ^V600E^BRAF and mTOR inhibition results in transcriptional control of TK1 protein levels in COLO 205 cells. (**A**) Western blot of COLO 205 cells treated with PP242 (250 nM) and increasing PLX4032. Similar to single agent PLX4032, p-MEK, but not p-ERK, was inhibited in a PLX4032-dependent manner. Consistent with mTORC1/mTORC2 inhibition, p-AKT Ser473, but not p-AKT Thr308, was inhibited. Unlike single agent PLX4032, which resulted in concentration-dependent activation of p-rpS6, combined treatment maintained p-rpS6 levels at essentially baseline levels except at the highest PLX4032 concentration. Similarly, DUSP6 levels were inversely related to p-ERK protein levels. With combined mTOR and ^V600E^BRAF blockade, p27 and TK1 protein levels were inversely correlated and dramatically affected by PLX4032 exposure. (**B**) Similarly, *TK1* mRNA was significantly reduced at PLX4032 concentrations as low as 10 nM. (**C**) Despite elevated p27 protein levels, *p27* mRNA was unaffected by combined mTOR-^V600E^BRAF inhibition.

To examine mTOR's role in TK1 regulation following ^V600E^BRAF inhibition, cultured COLO 205 cells were treated concomitantly with 250 nM pp242, a selective mTORC1/mTORC2 inhibitor [21] and increasing concentrations of PLX4032 for 48 hours (**Fig. 5**). Adding PP242 had little effect on PLX4032-dependent inhibition of p-MEK, yet effectively blocked activation of p-AKT at Ser473 and blunted the previously observed activation of p-rpS6 caused by PLX4032 exposure (compare to **Fig. 3A**). However, mTOR blockade was insufficient to attenuate p-ERK or p-AKT Thr308 levels in a PLX4032-dependent manner, nor to completely attenuate p-rpS6 levels (**Fig. 5A**). As with single agent *in vitro* studies, p-rpS6 activation correlated with a slight attenuation of DUSP6 levels and a slight elevation of p-ERK at higher PLX4032 concentrations. Nonetheless, combined inhibition of p-AKT Ser473 and p-MEK resulted in dramatically decreased TK1, at both the protein and mRNA level. In the presence of elevated p27, *TK1* mRNA levels fell dramatically, with significant reductions observed at PLX4032 concentrations as low as 10 nM (**Fig. 5A**). Furthermore, suggesting that AKT activity was responsible for post-translational modification and degradation of p27, in the presence of p-AKT Ser473 inhibition, p27 protein was dramatically elevated but *p27* mRNA was not (**Fig. 5C**).

**Figure 6:**
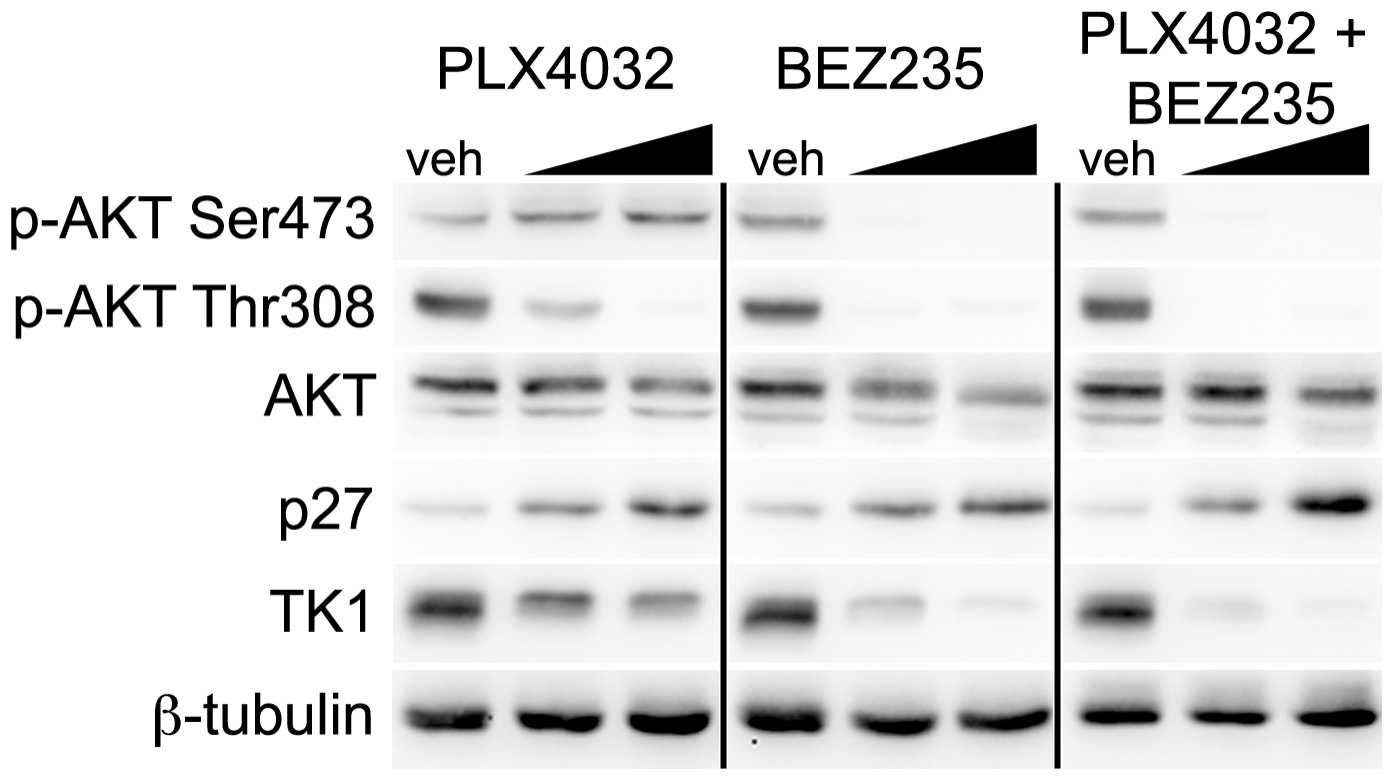
Dual pathway inhibition regulates TK1 protein levels and results in greater p27 protein levels than single agents alone. Single agent PLX4032 resulted in activation of p-AKT Ser473 following 24 hours of exposure at two concentrations (100 nM, 1 µM). The addition of the dual PI3K/mTOR inhibitor BEZ235 blocks p-AKT Ser473 activation and resulted in a greater increase in p27 protein levels and diminished TK1 protein levels.

Given that combined ^V600E^BRAF-mTOR inhibition was insufficient to attenuate p-rpS6, and coupled with the observation that PI3K activity and downstream signaling has been proposed as a mechanism of resistance to BRAF inhibition in colorectal cancer [22] and melanoma [23], [24], we evaluated the effects of dual PI3K-mTOR inhibition in COLO 205 cells in conjunction with ^V600E^BRAF inhibition (**Fig. 6**). COLO 205 cells were treated with either PLX4032, BEZ235, a small molecule dual inhibitor of PI3K and mTOR [25], or the combination for 24 hours. Indeed, inhibiting both mTOR activity, as measured by p-AKT Ser473, and PI3K activity, as measured by p-AKT Thr308, in the presence of PLX4032 resulted in greater p27 protein levels and decreased TK1 protein levels. These results prompted our evaluation of this combination *in vivo*.

### [^18^F]-FLT PET reflects inhibition of PI3K-mTOR activity in PLX4720-treated xenografts

**Figure 7:**
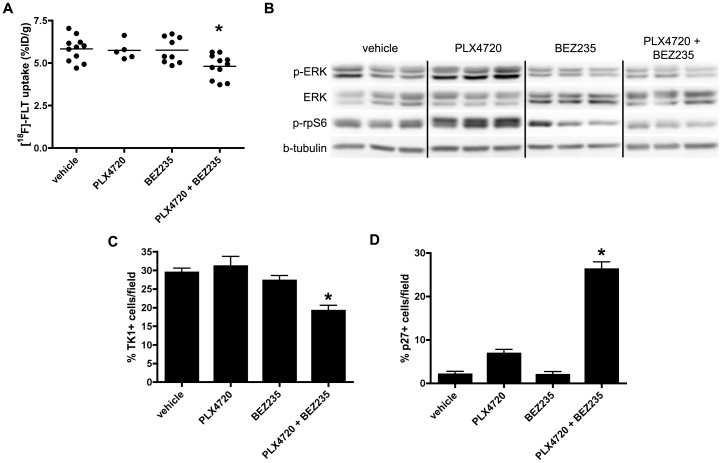
[^18^F]-FLT PET reflects BEZ235-dependent inhibition of PI3K/mTOR activity in PLX4720 treated COLO 205 xenografts. Xenograft-bearing mice were imaged with [^18^F]-FLT PET on treatment day 4. (**A**) [^18^F]-FLT uptake was diminished in the combination treatment cohort relative to vehicle (p = 0.0087), but not single agent PLX4720- or BEZ235-treated cohorts. (**B**) Western blot of xenograft tissue harvested immediately following imaging illustrated elevated p-ERK and p-rpS6 levels in PLX4720-treated mice. Combining PLX4032 with BEZ235 resulted in reduced p-ERK and p-rpS6 protein levels. (**C**) TK1 levels, as measured by IHC, were reduced only in the combination treatment group in agreement with [^18^F]-FLT PET. (**D**) Consistent with *in vitro* studies, diminished TK1 levels, and consequently [^18^F]-FLT PET, correlated with elevated p27 that was elevated only in the combination treated group.

To explore the effects of dual PI3K-mTOR inhibition on [^18^F]-FLT PET *in vivo*, we treated COLO 205 xenograft bearing mice daily with either vehicle, PLX4720, BEZ235, or in combination, and imaged the mice on treatment day 4. Strikingly, only the combination cohort exhibited decreased [^18^F]-FLT PET relative to vehicle-treated controls or single agent-treated cohorts (**Fig. 7A**). Western blot analysis of imaging-matched xenograft tissue revealed the anticipated increase of p-ERK levels and p-rpS6 levels in PLX4720 treated mice (**Fig. 7B**). However, combination treatment with PLX4720 and BEZ235 led to an overall reduction of p-ERK and p-rpS6 levels compared to vehicle-treated controls. In agreement with imaging, TK1 protein levels were reduced in the combination group only (**Fig. 7C**), although Ki67 immunoreactivity was reduced for all treatment groups relative to the vehicle-treated cohort (**Fig. S8**). Importatntly, and agreement with *in vitro* studies, combination treatment led to a significant increase in p27 protein levels, but not mRNA levels, compared to vehicle and single agent cohorts (**Fig. 7D**, **Fig. S9**). These results suggest that the primary determinant of reduced [^18^F]-FLT PET in this setting was inhibition of ^V600E^BRAF and PI3K-mTOR activity in conjunction with the rise in p27 protein levels.

## Discussion

Imaging biomarkers to both predict early response to targeted therapy was well as to predict resistance to treatment response are essential components of personalized medicine in oncology. Current clinical means of assessing treatment response by imaging are based upon the Response Evaluation Criteria in Solid Tumors (RECIST) guidelines [26]. Based upon changes in tumor size, RECIST does not take advantage of cellular and molecular information now available through contemporary imaging methodologies. As cellular and molecular changes can occur within hours of treatment and may precede changes in tumor volume, newer criteria, such as PERCIST [27], recognize the potential utility of [^18^F]-FDG PET to predict early response. However, as [^18^F]-FDG uptake in tissue reflects a broad range of metabolic processes, a more specific marker of cellular proliferation may better assess treatment response in targeted therapy [28].

[^18^F]-FLT has been extensively studied in small animal models of treatment response and in clinical trials. While many studies, including our own, have shown [^18^F]-FLT to be sensitive to treatment efficacy [4], [5], others have failed to show changes in [^18^F]-FLT tumor uptake despite effective treatment [3], [29]. We have extensively investigated the relationship between [^18^F]-FLT PET and proliferation, and have shown that [^18^F]-FLT PET does not necessarily reflect proliferation directly [6]. [^18^F]-FLT PET exhibits sensitivity to vital cellular processes such as activation of signaling pathways, as we demonstrate in this study.

In the context of EGFR inhibition in a wild-type KRAS colorectal cancer cell line we show that a lack of TK1 attenuation, and thus similar [^18^F]-FLT PET, in treated xenografts reflected PI3K-mTOR activity. Where PI3K-mTOR activity was elevated, [^18^F]-FLT PET did not reflect target inhibition and did not correlate with pharmacodynamic response. In contrast, when mTOR activity was abrogated, either genetically or pharmacologically, TK1 levels were attenuated, which, in turn, lead to decreased [^18^F]-FLT PET. Here, [^18^F]-FLT PET provides clinically important information that is unavailable through [^18^F]-FDG PET imaging. We further studied this phenomenon in a different therapeutic setting, the inhibition of ^V600E^BRAF in a ^V600E^BRAF mutant colorectal cancer cell line. While inhibition of mTOR signaling in EGFR blockade in wild-type KRAS was sufficient to control TK1, both the PI3K and mTOR pathways served to maintain TK1 protein levels by transcriptional and post-translational mechanisms during pro-survival signaling during ^V600E^BRAF inhibition. As such, [^18^F]-FLT PET may aid the elucidation of the mechanisms of resistance to targeted therapies which are relevant in colorectal cancer [30], and potentially other solid tumors such as melanoma [31].

In many cases tumors may initially respond to targeted therapy only to recur with more aggressive phenotypes, notably as after ^V600E^BRAF inhibition in melanoma [31]–[33]. It is therefore important clinically to detect activation of resistance pathways and to predict possible recurrence. This study demonstrates that [^18^F]-FLT may serve as an early PET biomarker that may be sensitive to activation of pro-survival mechanisms that may predict tumors that are more likely to resist treatment and ultimately may be more prone to recurrence. It is notable that [^18^F]-FDG PET was insufficient to observe the activity of the pro-survival signals detected by [^18^F]-FLT PET. Therefore, we envision a new role for [^18^F]-FLT PET in the setting of predicting response to targeted therapy.

## Supporting Information

Figure S1 **Elevated p27 protein levels observed following cetuximab exposure in DiFi cells are not transcriptionally induced.** No statistically significant change in *p27* was observed in DiFi cells treated with raptor siRNA or scrambled RNA when treated with either 0.5 µg/mL or 5.0 µg/mL cetuximab.
(TIF)

Figure S2 **Elevated p27 protein levels observed following cetuximab treatment in DiFi xenografts are not transcriptionally induced.** No statistically significant change in *p27* levels was observed in DiFi xenografts treated with either 20 mg/kg or 40 mg/kg cetuximab relative to vehicle-treated xenografts.
(TIF)

Figure S3 **Cetuximab treatment attenuates p-rpS6 immunoreactivity at 40 mg/kg but not 20 mg/kg.** No difference in p-rpS6 immunoreactivity was observed between vehicle-treated controls and DiFi tumor xenografts treated with 20 mg/kg cetuximab (p = 0.9743). When treated with 40 mg/kg cetuximab, DiFi tumor xenografts exhibit reduced p-rpS6 immunoreactivity compared to vehicle-treated tumors (p = 0.0334).
(TIF)

Figure S4 **Cetuximab treatment attenuates Ki67 immunoreactivity at both 20 mg/kg and 40 mg/kg.** Ki67 IHC was reduced at both 20 mg/kg (p<0.0001) and 40 mg/kg (p<0.0001) cetuximab compared to vehicle-treated controls.
(TIF)

Figure S5 **[^18^F]-FDG PET does not reflect PI3K-mTOR signaling in cetuximab-treated DiFi xenografts** DiFi tumor xenografts were imaged on day 7 of a 20 mg/kg or 40 mg/kg cetuximab treatment regimen. In contrast to [^18^F]-FLT PET, [^18^F]-FDG PET was similarly reduced at both the 20 mg/kg (p = 0.0286) and 40 mg/kg (p = 0.0286) dose levels.
(TIF)

Figure S6 **Inhibition of MAPK-pathway activity in COLO 205 cells following exposure to PLX4032 for 2 hours.** ^V600E^BRAF downstream effectors p-MEK and p-ERK were similarly inhibited following 2 hours PLX4032 exposure.
(TIF)

Figure S7 **Relative inhibition of ^V600E^BRAF downstream effectors following 24 hour exposure of PLX4032 in COLO 205 cells.** Cells were collected at 24 hours following treatment with 10 nM, 100 nM, 500 nM, 1 µM, or 5 µM PLX4032.
(TIF)

Figure S8 **Ki67 is reduced in COLO 205 xenografts treated with PLX4720, BEZ235, as well as the combination.** Ki67 immunostaining was significantly reduced in all treatment regimens in COLO 205 xenografts (p<0.0001) compared to vehicle-treated xenografts.
(TIF)

Figure S9 ***p27*** **mRNA is not affected by PLX4720, BEZ235, or combination treatment in COLO 205 tumors.** No change in *p27* mRNA levels was observed in any treatment regimen compared to vehicle-treated xenografts.
(TIF)
