# Supplementary figures and images for: 3′-Deoxy-3′-[18F]-Fluorothymidine PET Imaging Reflects PI3K-mTOR-Mediated Pro-Survival Response to Targeted Therapy in Colorectal Cancer
Source: PLoS One. 2014 Sep 23;9(9):e108193. doi: 10.1371/journal.pone.0108193 (PMC4172755; doi:10.1371/journal.pone.0108193)

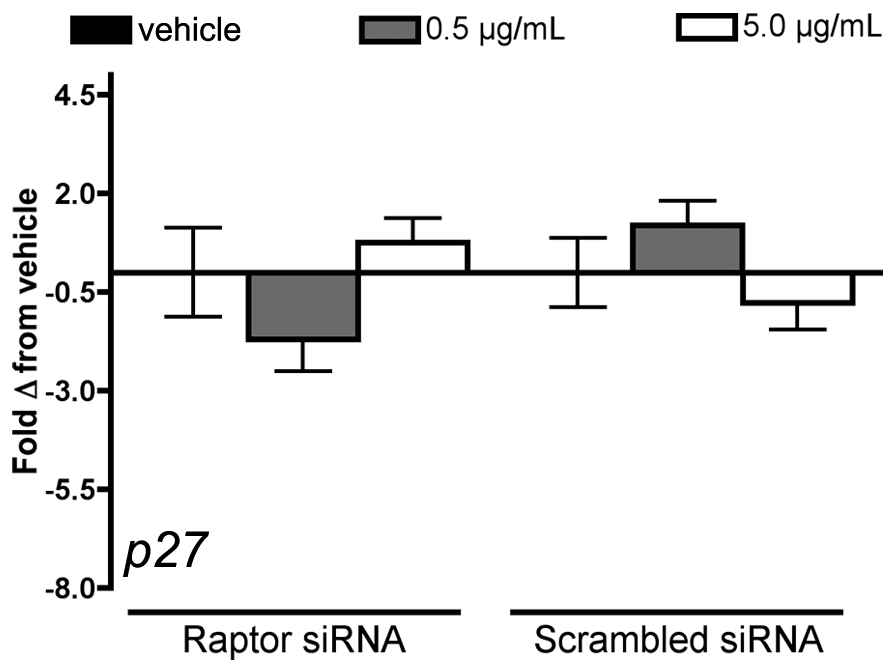

Supplement: Figure S1 — Elevated p27 protein levels observed following cetuximab exposure in DiFi cells are not transcriptionally induced. No statistically significant change in p27 was observed in DiFi cells treated with raptor siRNA or scrambled RNA when treated with either 0.5 µg/mL or 5.0 µg/mL cetuximab. (TIF) [file pone.0108193.s001.tif]

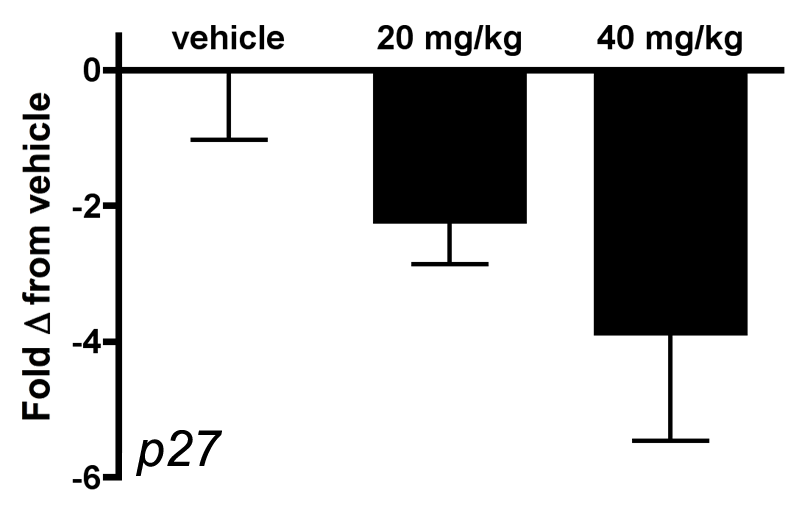

Supplement: Figure S2 — Elevated p27 protein levels observed following cetuximab treatment in DiFi xenografts are not transcriptionally induced. No statistically significant change in p27 levels was observed in DiFi xenografts treated with either 20 mg/kg or 40 mg/kg cetuximab relative to vehicle-treated xenografts. (TIF) [file pone.0108193.s002.tif]

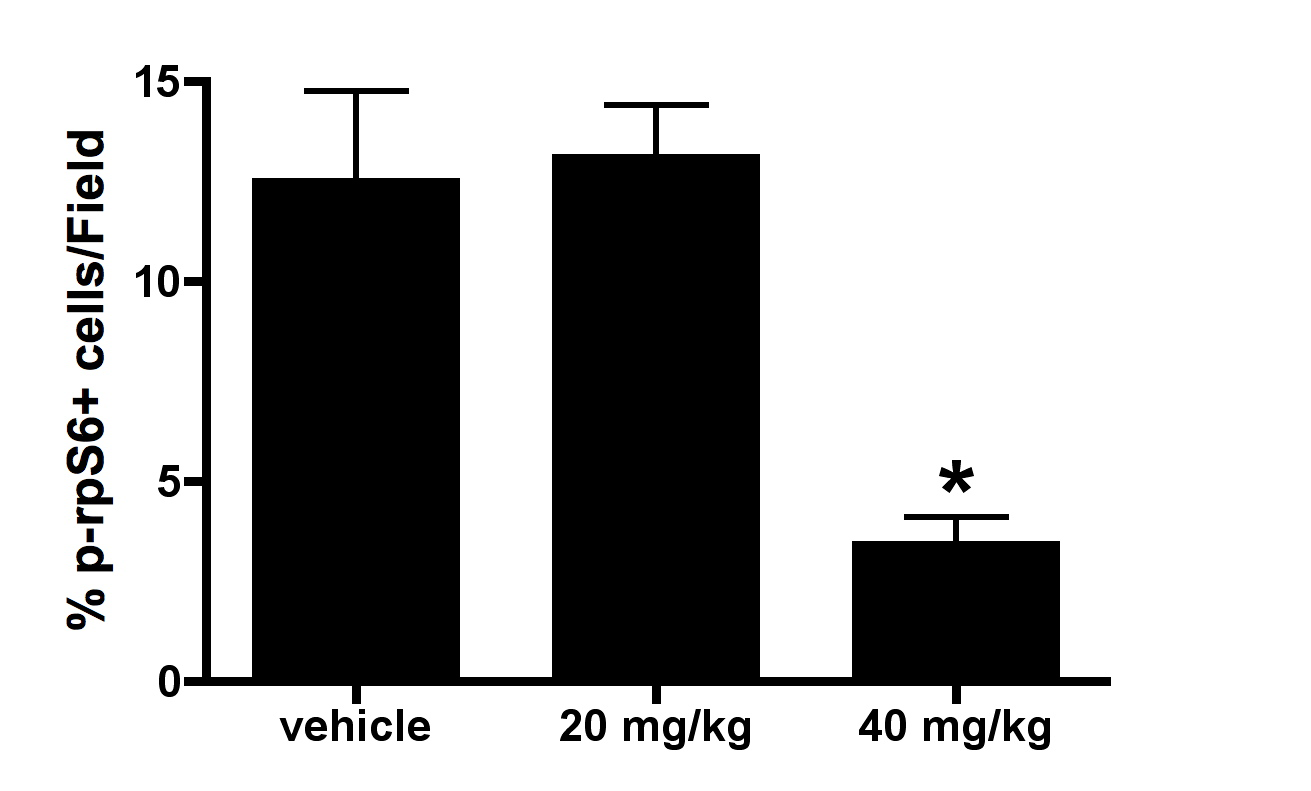

Supplement: Figure S3 — Cetuximab treatment attenuates p-rpS6 immunoreactivity at 40 mg/kg but not 20 mg/kg. No difference in p-rpS6 immunoreactivity was observed between vehicle-treated controls and DiFi tumor xenografts treated with 20 mg/kg cetuximab (p = 0.9743). When treated with 40 mg/kg cetuximab, DiFi tumor xenografts exhibit reduced p-rpS6 immunoreactivity compared to vehicle-treated tumors (p = 0.0334). (TIF) [file pone.0108193.s003.tif]

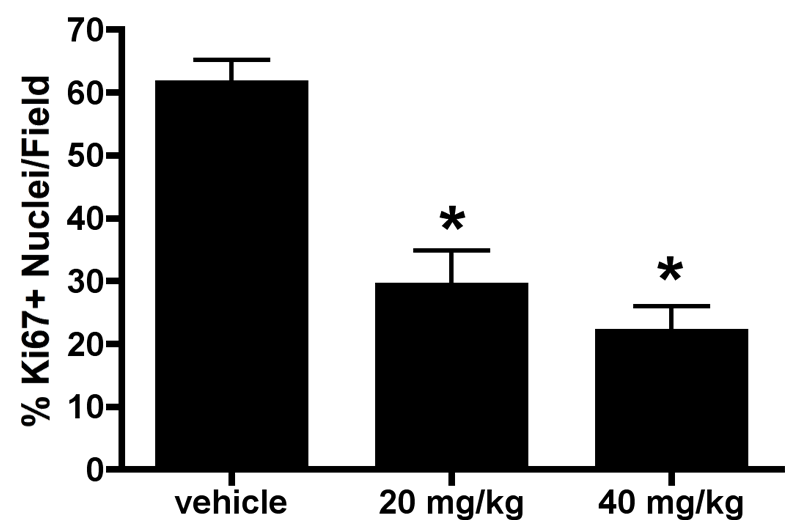

Supplement: Figure S4 — Cetuximab treatment attenuates Ki67 immunoreactivity at both 20 mg/kg and 40 mg/kg. Ki67 IHC was reduced at both 20 mg/kg (p<0.0001) and 40 mg/kg (p<0.0001) cetuximab compared to vehicle-treated controls. (TIF) [file pone.0108193.s004.tif]

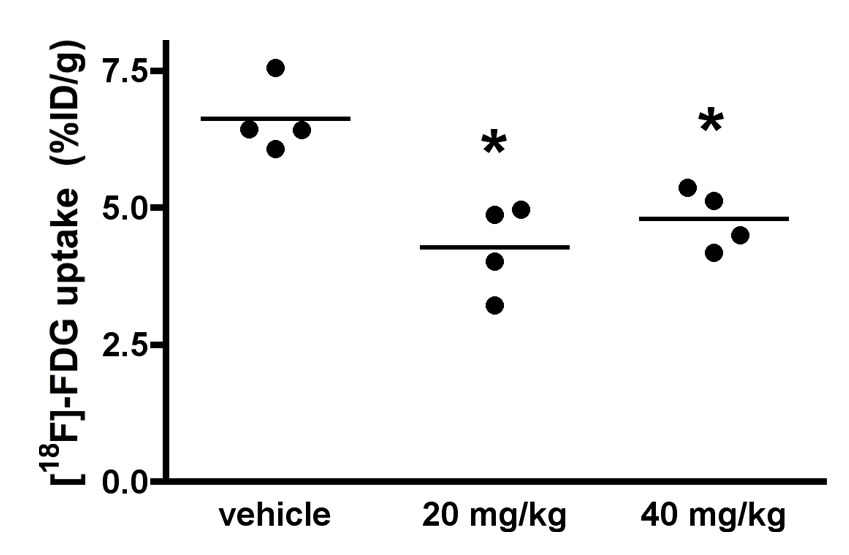

Supplement: Figure S5 — [18F]-FDG PET does not reflect PI3K-mTOR signaling in cetuximab-treated DiFi xenografts DiFi tumor xenografts were imaged on day 7 of a 20 mg/kg or 40 mg/kg cetuximab treatment regimen. In contrast to [18F]-FLT PET, [18F]-FDG PET was similarly reduced at both the 20 mg/kg (p = 0.0286) and 40 mg/kg (p = 0.0286) dose levels. (TIF) [file pone.0108193.s005.tif]

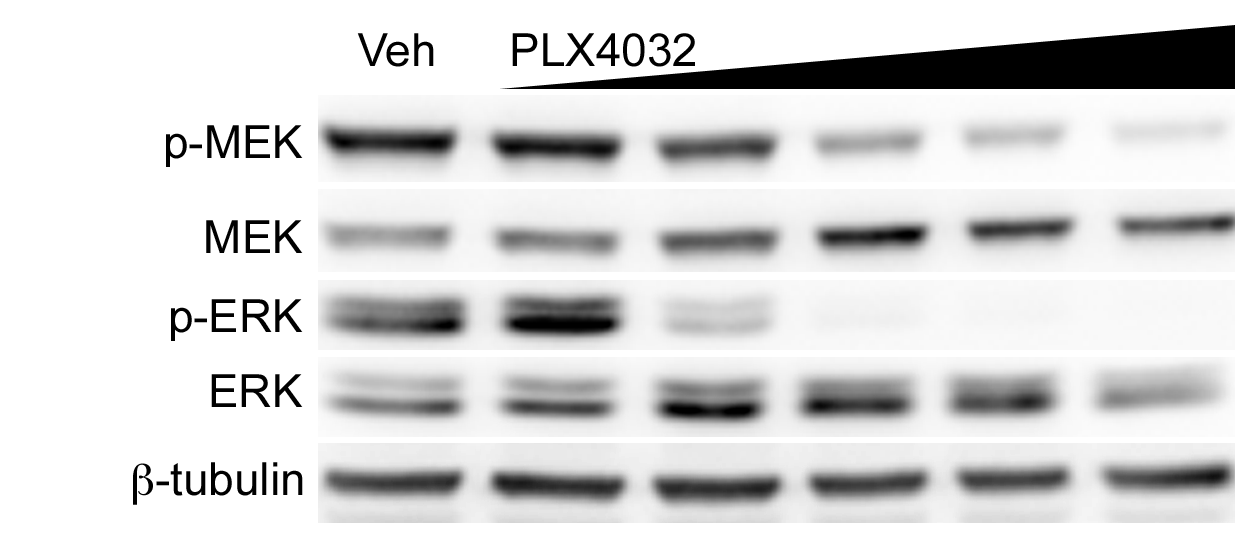

Supplement: Figure S6 — Inhibition of MAPK-pathway activity in COLO 205 cells following exposure to PLX4032 for 2 hours. V600EBRAF downstream effectors p-MEK and p-ERK were similarly inhibited following 2 hours PLX4032 exposure. (TIF) [file pone.0108193.s006.tif]

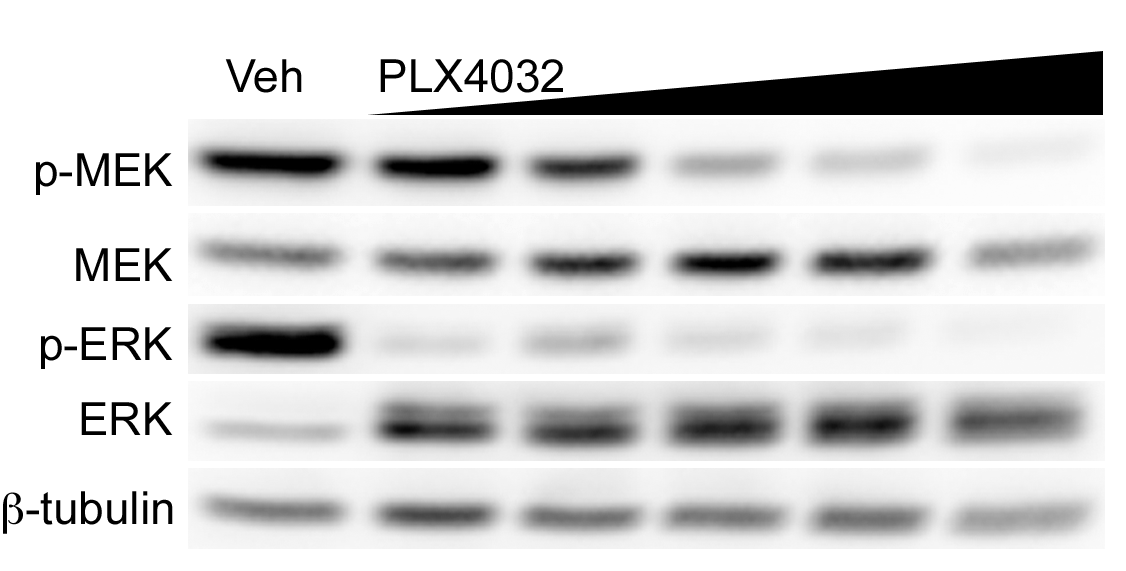

Supplement: Figure S7 — Relative inhibition of V600EBRAF downstream effectors following 24 hour exposure of PLX4032 in COLO 205 cells. Cells were collected at 24 hours following treatment with 10 nM, 100 nM, 500 nM, 1 µM, or 5 µM PLX4032. (TIF) [file pone.0108193.s007.tif]

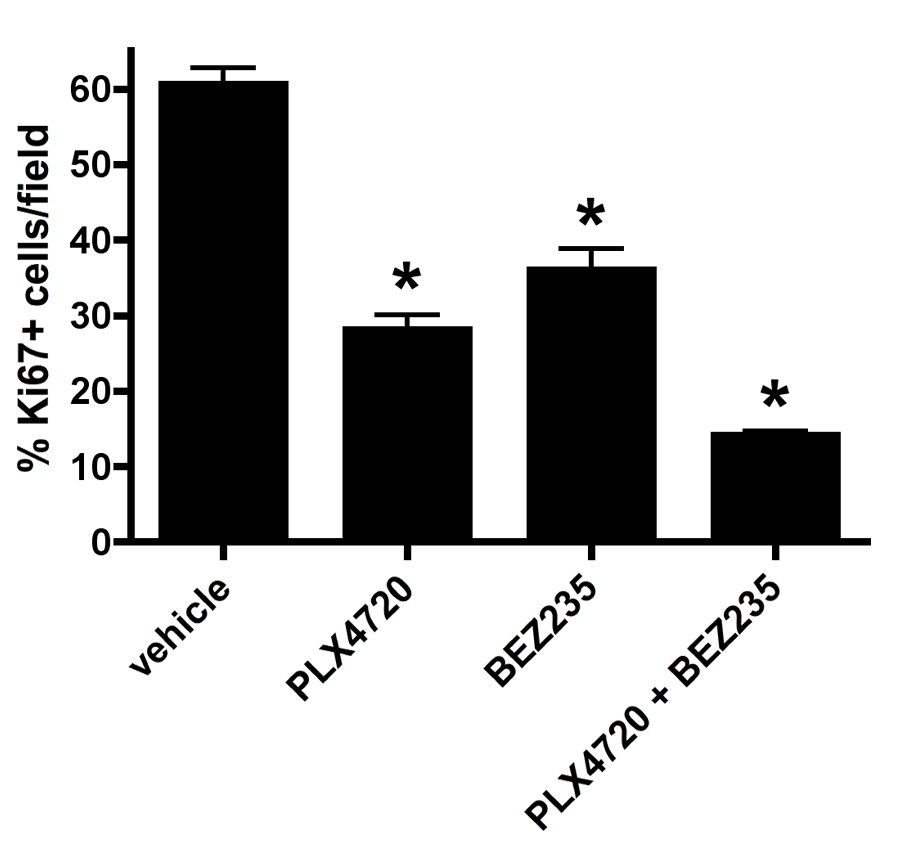

Supplement: Figure S8 — Ki67 is reduced in COLO 205 xenografts treated with PLX4720, BEZ235, as well as the combination. Ki67 immunostaining was significantly reduced in all treatment regimens in COLO 205 xenografts (p<0.0001) compared to vehicle-treated xenografts. (TIF) [file pone.0108193.s008.tif]

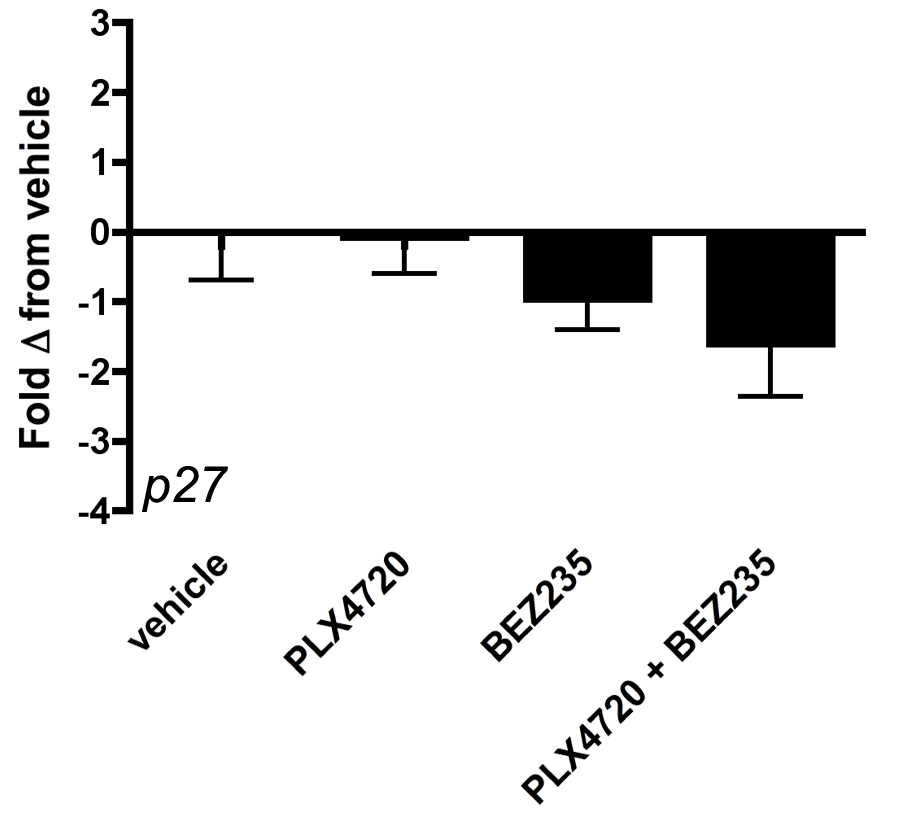

Supplement: Figure S9 — p27 mRNA is not affected by PLX4720, BEZ235, or combination treatment in COLO 205 tumors. No change in p27 mRNA levels was observed in any treatment regimen compared to vehicle-treated xenografts. (TIF) [file pone.0108193.s009.tif]
